# Supplementary material for: Rapid turnover of life-cycle-related genes in the brown algae
Source: Genome Biol. 2019 Feb 14;20:35. doi: 10.1186/s13059-019-1630-6 (PMC6374913; doi:10.1186/s13059-019-1630-6)
Supplement: Supplementary file 3 — Protein alignments, corresponding Gblocks html file and CDS sequences for the lineage conserved gametophyte-biased genes. (ZIP 2109 kb) [file 13059_2019_1630_MOESM3_ESM.zip › Con_GA_alignments/Ec-23_000730.aa_ali.fasta-gb.htm]

Ec-23\_000730.aa.fasta.trans.aa\_ali.fasta


## Gblocks 0.91b Results

Processed file: **Ec-23\_000730.aa.fasta.trans.aa\_ali.fasta**  
Number of sequences: **2**  
Alignment assumed to be: **Protein**  
New number of positions: **511** (selected positions are underlined in blue)

```
                         10        20        30        40        50        60
                 =========+=========+=========+=========+=========+=========+
Ec-23_000730     ------------------------------------------MHGMNDEEQLEQSHQDCL
MSTRG.52840.1    MVDVASIVKLAFVLSQVPQTVKINRGQCQLLVDRITSLAGHLERMQNVHEKLERSHEECI
                                                               ##############


                         70        80        90       100       110       120
                 =========+=========+=========+=========+=========+=========+
Ec-23_000730     VRAHAVLVECQEFMRSFEDGKWMVKALKKDKHAETFQVLHESLTYIFQNLHLEIDYHSLK
MSTRG.52840.1    ARAHGVVLECEEFMRSFEDGMWMVKALKREVHREKFSELHESLTYVFQVLHLEIDYHSLK
                 ############################################################


                        130       140       150       160       170       180
                 =========+=========+=========+=========+=========+=========+
Ec-23_000730     KQSAQAEMRDVAHVEDQLASVAANMEVLVF----------------------LRMPEKTI
MSTRG.52840.1    KRSIDAEMRDVAHMEDQLAGIAANMEKLEQSVAALTDESKKHHDDIMAALRNVRVYRKTL
                 ############################                                


                        190       200       210       220       230       240
                 =========+=========+=========+=========+=========+=========+
Ec-23_000730     SWK------ATVKLPRGLGHPPHTRMVPKGSMDKKRRTKDATGAIHPQARLTP-------
MSTRG.52840.1    GQDQLDSVLATAKEDERSEVEKESDVSASSSPAARVRSMAAQDAVEPPSCLQSSTLECRE
                                               #####################         


                        250       260       270       280       290       300
                 =========+=========+=========+=========+=========+=========+
Ec-23_000730     -------------------------GRPPKPGVNATAVQSVASKAVNGRALGPSLHIKPY
MSTRG.52840.1    AEEHEDRKRSQNSIVISGNVSSSSSDRPPKSSIIKSGLAANAS--TNARTLGSSLHTR-L
                                                               ##########    


                        310       320       330       340       350       360
                 =========+=========+=========+=========+=========+=========+
Ec-23_000730     SQSYGVG--LAAGVMAGGWLADAGLASGASAGDGGAKRGEYEVSFGEGPMGMSLKNVDAR
MSTRG.52840.1    GQSLYIGGRLAVGMVEESRLAHTSSRTESGAGDGGAKRGEYEVSFGEGQMGMSLKNVEAR
                          ############         ##############################


                        370       380       390       400       410       420
                 =========+=========+=========+=========+=========+=========+
Ec-23_000730     AEVSKTEQGGAAAAAGVRPGDNVSGINGRIPLGYTQVMGTLPTTPRPVRICFTRGQGSTI
MSTRG.52840.1    AEVSKVEPGGSAHAAGVRPADSVSGINGRIPLGYTQVMSILPTAPRPVRICFVRGQRTTL
                 ############################################################


                        430       440       450       460       470       480
                 =========+=========+=========+=========+=========+=========+
Ec-23_000730     SPGGPGKKSHASTWASNAVTAGVHHPAGDKGVLLEIYAQASLSSTTWVRSQGWDELPPP-
MSTRG.52840.1    SPGSKEGRTHASTWASNAVTAGVHHPAGDRGVLLEMYAKASFSQTGWIRSQGWDGLPMPP
                 ########################################################### 


                        490       500       510       520       530       540
                 =========+=========+=========+=========+=========+=========+
Ec-23_000730     ----ETEDCAQAEGTTKENRSGSDIGLERWSGVRIGRKNKVVEIRLPGNGMVGVLPASTG
MSTRG.52840.1    PLPGSEKETSKGEAATNESGYADEVPARHFSGVRVGRGEKVIEIRLPGNDMHGVLPPSVG
                                               ##############################


                        550       560       570       580       590       600
                 =========+=========+=========+=========+=========+=========+
Ec-23_000730     SLDELRILELRENRLEGNIPESLRYLQKLTKVDLAHNLLEGSVPGGDARWIGSMSVLLLQ
MSTRG.52840.1    SLDELRALELRENRLEGNIPESLRYLHKLTKIDLAHNMFEGSVPGGDARWIKSMSVILLN
                 ############################################################


                        610       620       630       640       650       660
                 =========+=========+=========+=========+=========+=========+
Ec-23_000730     YNKLSGHIPSTLGRLVNLTQLDLSFNQLSGNIPSELSNARALEVLSLCNNVLIGPVPESF
MSTRG.52840.1    NCRLSGHIPSTLGRLLNLTHLDLSFNALSGSIPPELSAARALEILNVSNNTLNGPLPQTL
                 ############################################################


                        670       680       690       700       710       720
                 =========+=========+=========+=========+=========+=========+
Ec-23_000730     GGLTNLKVLNASNNKLAGPLPQGLGRLTRLEVLSLQHNLLNGSIPDDTLSSDSLVFVDVS
MSTRG.52840.1    GGLTRLKILNAANNQLVGPLTKDLGQLTCLEVLSLANNRLSGNIPHEAFSSPCLVMVDMA
                 ############################################################


                        730       740       750       760       770       780
                 =========+=========+=========+=========+=========+=========+
Ec-23_000730     FNALEGKV------------HVETLKS-----------GIVVVQDGPAHHDSVVGPPRPS
MSTRG.52840.1    SNGLEGKIPNEICTRLANLTHLDISKNSLSGELPSSIGSICQLKKLNLEKNSMEGALPTS
                 #######                                                     


                        790       800       810       820       830       840
                 =========+=========+=========+=========+=========+=========+
Ec-23_000730     NV----------------------------------------------------------
MSTRG.52840.1    LAECKALEDLNLSNNLLVGPLLHIHWENFCSLEHLLLANNKLEGALPASFGSLASLVSLD
                                                                             


                        850       860       870       880       890
                 =========+=========+=========+=========+=========+========
Ec-23_000730     ---------------------------------------------------------H
MSTRG.52840.1    ISKNQIAGSIPRELHELTGLKLLDISGNKIASSGLLPRTSFFSGKRTLKKKLSACDVR
```

```
Parameters used
Minimum Number Of Sequences For A Conserved Position: 2
Minimum Number Of Sequences For A Flanking Position: 2
Maximum Number Of Contiguous Nonconserved Positions: 8
Minimum Length Of A Block: 10
Allowed Gap Positions: None
Use Similarity Matrices: Yes
```

```
Flank positions of the 6 selected block(s)
Flanks: [47  148]  [211  231]  [287  296]  [310  321]  [331  479]  [511  727]  

New number of positions in Ec-23_000730.aa.fasta.trans.aa_ali.fasta-gb:  511  (56% of the original 898 positions)
```
